# Effects of *Scrophularia ningpoensis* Hemsl. on Inhibition of Proliferation, Apoptosis Induction and NF-κB Signaling of Immortalized and Cancer Cell Lines

**DOI:** 10.3390/ph5020189

**Published:** 2012-02-14

**Authors:** Xiao Shen, Tolga Eichhorn, Henry Johannes Greten, Thomas Efferth

**Affiliations:** 1 Department of Pharmaceutical Biology, Institute of Pharmacy and Biochemistry, Johannes Gutenberg University, Staudinger Weg 5, Mainz 55128, Germany; 2 Heidelberg School of Chinese Medicine, Heidelberg 69126, Germany; 3 Biomedical Sciences Institute Abel Salazar, University of Porto, Porto 4000, Portugal

**Keywords:** apoptosis, microarrays, pharmacogenomics, pharmacognosy, phytotherapy, signaling pathways

## Abstract

*Scrophularia ningpoensis* has been used in China for centuries as a herbal tea to treat various diseases. Based on the numerous animal studies on its pharmaceutical effects and the long time clinical experiences, we studied the molecular and cellular mechanism underlying the bioactivity of aqueous extract of *Scrophularia* and its isolated compounds. Seven isolated compounds, unlike *Scrophularia* extract, failed to induce cytotoxicity on HaCaT cells, but their combination improved the effect of extract. Tumor cell line selectivity was not observed, when we studied its cytotoxic effect on melanoma cell lines. The apoptotic and anti-inflammatory effects of *Scrophularia* extract have been demonstrated on HaCaT cells. The extract induced those effects potentially through affecting the MAPK pathway and inhibition of the NF-κB pathway, Microarray-based bioinformatical analyses on the compound acetoside from *Scrophularia* revealed a gene expression profile which confirmed our findings with the extract on proliferation inhibition, anti-inflammation and apoptosis. With DNA alkylation as major proposed mechanism of action, we assume acetoside as one of the active compounds in *Scrophularia*.

## 1. Introduction

The genus *Scrophularia* consists of about 300 species, many of which have been used for medical care in folk medicine since ancient times. The dried roots of* Scrophularia ningpoensis* Hemsl. (*Radix Scrophulariae ningpoensis*, RSN, Ningpo figwart, *Scrophulariaceae*) are commonly used as tea in Traditional Chinese Medicine [1]. Known for its antipyretic and anti-inflammatory effects, RSN is widely used for the treatment of laryngitis, fever, swelling, constipation and neuritis [2,3]. Small doses of RSN act as a heart tonic, while higher doses rather suppress heart function.

With growing interest for Chinese phytotherapy research in Western countries, the chemical compounds of RSN and their medical function have been investigated quite intensely during the past decade. Many classes of secondary metabolites have been found in RSN, -of which iridoids and phenylpropanoids, represented by harpagide, harpagoside and angoroside C, were believed to be the main biologically active compounds. Harpagide is also used as a reference substance for RSN identification. Besides iridoids and phenylpropanoids, other ingredients such as phytosterols, phenolic acids, flavonoids, saponins and naphtha have also been detected in the plant [4,5]. The main components detected so far are summarized in Table 1.

**Table 1:** Main chemical composition and their approximate amount in RSN [1,4,5,6,7,8,9].

Iridoids	Phenylpropanoid	Phytosterol	Organic acids
Harpagide 12.5 mg/kg	Sibirioside A 8 mg/kg	β-Sitosterol 12 mg/kg	Cinnamic acid 14 mg/kg
Harpagoside 20 mg/kg	Cistanoside F 7 mg/kg	Daucosterol	Ferulic acid
Galactopyranosylharpagoside	Angoroside C 2 mg/kg		Butane diacid
Feruloyharpagide 1 mg/kg	Cistanoside D 6 mg/kg		
Hydroxycinnamoylharpagide	Acetoside 12 mg/kg		
Acetylharpagoside	Decaffeoylacetoside 4 mg/kg		
Aucubin 3 mg/kg	Ningposide A 5 mg/kg		
Geniposide	Ningposide B 1.5 mg/kg		
Catapol	Ningposide C 3 mg/kg		
Methylcatapol 33 mg/kg	Ningposide D 6 mg/kg		
Scropolioside A			
Scrophuloside B4 6 mg/kg	**Triterpenoid saponins**	**Sugars**	**Terpenes**
Iridoidlacton	Ursolic acid 10 mg/kg	Fructose	Cryptomeriol
Ningpogenin	Asparagines	Sucrose	
Ningpogoside A	Triterpenoid saponins	Glucose	
Ningpogoside B			

Broad pharmacological activities of RSN extract and many of its compounds have mostly been studied in animal experiments. Sun *et al*. described the hepatoprotective effects of phenylpropanoids from RSN aminotransferases [6]. Fast repair of oxidized OH radical adducts of dAMP and dGMP by angoroside C and acetoside generated by RSN was demonstrated [10], suggesting an anti-oxidative effect of *Scrophularia ningpoensis*. The plant also acts as immuno-modulator stimulating B cells [11]. Angoroside C and acetoside revealed anti-inflammatory activities in rats [12]. Other activities such as anti-angiogenesis [13], neuroprotection [14], as well as anti-depressant [15], ant-amnestic [16], cardiovascular [17], cytotoxic [18], anti-tumor [19] properties also have been reported. 

Despite numerous investigations demonstrating the bioactivity of RSN, the molecular and cellular mechanisms of action are still poorly understood. For this reason, we investigated the role of NF-кB and its signaling pathways for the cytotoxic activity of RSN towards HaCaT and melanoma cells. Because NF-кB represents an important mediator of inflammatory and apoptotic signals, we hypothesized that this transcription factor might also be a major player for the cytotoxic activity of RSN.

## 2. Experimental Section

### 2.1. Chemicals

**Figure 1:**
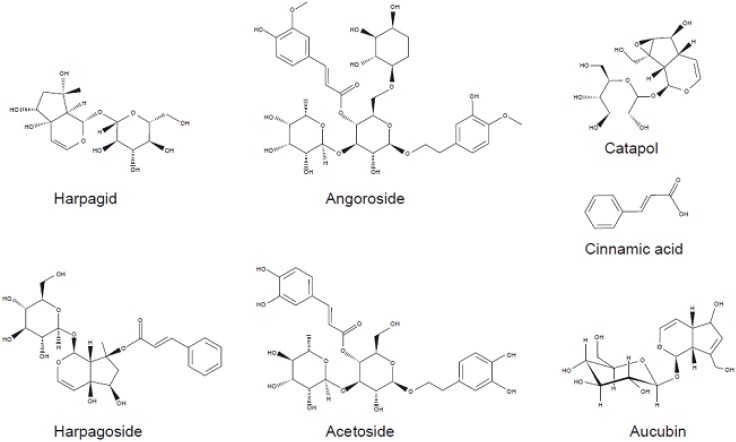
Chemical structures of phytochemicals from *Scrophularia ningpoensis* Hemsl.

Harpagid (XS-6, C_15_H_24_O_10_), harpagoside (XS-7, C_24_H_30_O_11_), aucubin (C_15_H_22_O_9_), catpol (C_15_H_22_O_10_), acetoside (C_29_H_36_O_15_) and trans-cinnamic acid (C_9_H_8_O_2_) purchased from ChromaDex LGC Standards (Wesel, Germany), and angoroside C(C_36_H_48_O_19_) purchased from AXXORA (Lörrach, Germany) were commercially available compounds from *Scrophularia ningpoensis*. The chemical structures are shown in Figure 1. All compounds were diluted to 100 mM in dimethyl sulfoxide (DMSO) for use. 

### 2.2. Aqueous Herbal Extract

*Radix Scrophulariae* *ningpoensis* (*xuan shen*, HerbaSinica Hilsdorf GmbH, Rednitzhembach, Germany; batch no. 060601E087, harpagoside content: 0.13%) was cleaned under distilled water, dried, cut into small pieces, diluted in 10 times (w/w) distilled water and boiled for 60 min. The solute was percolated through filter paper (Whatman, pleated filter grade 597½, 4–7 µm) and then sterilized by filtering through a 0.22 µm pore filter (Corning Incorporated, Berlin, Germany). The resulting extract was concentrated to one tenth, adjusted to a concentration of 1 mg/mL, and stored in aliquots at −20 °C.

### 2.3. Cell Culture

The HaCaT cell line, a spontaneously transformed non-tumorigenic human keratinocyte line [20] was kindly given by Ronny Enk (Heidelberg, Germany), and was cultured in D-MEM medium (Gibco), supplemented with 10% fetal bovine serum (FBS), 100 U/mL penicillin G and 100 µg/mL streptomycin, in an atmosphere of 5% CO_2_ at 37 °C. The cells were passaged 3–4 days before experiments, until reaching confluent state of 50–80%.

Three malignant melanoma cell lines, Colo38 [21], SK-Mel-28 [22], and MRI-221 [23], were kindly provided by Elisabeth F. Groene (German Research Center, Heidelberg, Germany). The SK-Mel-28 was cultured at the same condition as HaCaT cell line. Colo38 and MRI-221 cells were grown in suspension in RPMI 1640 medium (Biochrom AG, Berlin, Germany) added with 10% FCS, 2 mM L-glutamine 100 U/mL penicillin G and 100 µg/mL streptomycin in a humidified atmosphere of 5% CO_2_ at 37 °C.

### 2.4. XTT Proliferation Assay

The activities of the S*crophularia* extract or derived compounds on proliferation were determined by means of the Cell ProliferationKit II (Roche Diagnostics, Mannheim, Germany). This test is based on the cleavage of the yellow 2, 3-bis[2-methoxy-4-nitro-5-sulfophenyl]-2H-tetrazolium-5-carboxanilide inner salt (XTT) by ubiquitous dehydrogenases leading to the formation of an orange formazan dye. The amount of dye is commensurate to the number of metabolic active cells. The procedure has been described by us in detail [24].

The cytotoxic effect of the treatment was determined as percentage of viability compared to untreated cells: Absorbance of untreated cells – Absorbance of medium background


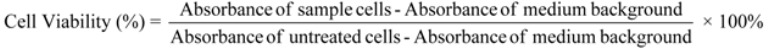


Origin Pro 7.5 was used for data analysis.

### 2.5. Immunofluorescence

HaCaT cells were seeded on cover slips laying in each well of 24 well plate three days before experiment. Cells were treated with different concentrations of *Radix Scrophularia ningpoensis* extract for 1 h at 37 °C, 5% CO_2_ in a humidified atmosphere. Ten nanograms of recombinant human tumor necrosis factor-α (TNF-α, Promega GmbH, Mannheim, Germany) were applied for 30 min to stimulate an inflammatory state in HaCaT cells. Cells were washed three times with phosphate buffered saline (PBS), fixed with 4% paraformaldehyde for 15 min at 4 °C and washed three times with 50 mM NH_4_Cl/PBS for 10 min. Cells were permeabilized with 0.1% Triton X-100 (in PBS) for 3 min and blocked for 30 min in 0.2% gelatine/PBS. Primary antibody (NF-κB p65 (F-6) purified mouse IgG, sc-8008, Santa Cruz Biotechnology, Inc., Heidelberg, Germany) was diluted 1:250 in 0.2% gelatine/PBS and incubated for 60 min. After three washing steps with 0.2% gelatine/PBS, cells were simultaneously incubated in the dark for 60 min with a secondary antibody (CyTM 3-conjugated Donkey anti-mouse IgG, Jackson ImmunoResearch Laboratories, Inc., West Grove, PA, USA) and Hoechst 33342 nuclear stain (Molecular Probes Inc., Eugene, OR, USA), both diluted 1:1,000 in 0.2% gelatine/PBS. Cover slips were subsequently washed with PBS and mounted with Mowiol (Calbiochem-Novabiochem, Bad Soden/Taunus, Germany) (50% glycerol in PBS) for observation. Image acquisition was achieved by using an Olympus fluorescent microscope (Olympus Imaging America Inc., Center Valley, PA, USA).

### 2.6. Western Blot

HaCaT cells were incubated with *Radix Scrophulariae ningpoensis* extract for 1 h and subsequently stimulated with 10 ng TNF-α for 30 min. After collecting cells in cold PBS, cytosolic and nuclear cell fractions were separated (first buffer: 10 mM Hepes pH 7.9, 10 mM KCl, 0.1 mM EDTA, 1.5 mM MgCl_2_, 0.1% NP4O, 1 m MDTT; second buffer: 20 mM Hepes pH 7.9, 420 mM NaCl, 0.1 mM EDTA, 1.5 mM MgCl_2_, 25% glycerol, 1 m MDTT, 0.5 m MPMSF). Aliquots of cell fractions, which were normalized as to total protein content using Bradford Protein Assay (Bio-Rad Laboratories, Munich, Germany), were separated by SDS-PAGE and transferred to nitrocellulose membranes. Membranes were blocked (5% milk powder in 1× TBST buffer) and incubated with following antibodies: NF-κB p65 (F-6) purified mouse IgG (Santa Cruz Biotechnology, Inc.), p44/42 MAPK (Erk1/2) rabbit monoclonal antibody (mAb), SAPK/JNK (56G8) rabbit mAb, p38 MAPK rabbit mAb, Phospho-p38 MAPK (Thr180/Tyr182) rabbit mAb, Phospho-p44/42 MAPK (Erk1/2) (Thr202/Tyr204) rabbit mAb and Phospho-SAPK/JNK (Thr183/Tyr185) (81E11) rabbit mAb (all purchased from Cell Signaling Technology^®^ Inc., Danvers, MA, USA). Horseradish peroxidase-conjugated secondary antibodies were anti-rabbit IgG (Cell Signaling Technology) or anti-mouse IgG (Santa Cruz, 1:10,000). Reactions were hen detected by chemiluminescence using peroxidase-conjugated antibodies and visualized using the Enhanced Chemiluminescence Reagents (ECL System, Amersham Biosciences, Freiburg, Germany). Immunoreactive bands on autoradiography films were scanned (Epson GT 9600; Epson, Tokyo, Japan) or detected by universal hood II (Bio-Rad Laboratories).

### 2.7. Detection of Apoptosis

HaCaT cells were seeded on 10 cm diameter Petri dishes three days in advance of experiments, until they reached 50–70% confluence. Cells were incubated with concentration-gradient-extract in time intervals of 6, 24, 48, 72 and 96 h, and both flowing cells and adherent cells were collected. Cells were washed with ice-cold PBS (+1 mM EDTA), followed by fixation with 70% ethanol for at least 2 h at −20 °C. After washing in PBS (+1 mM EDTA) for two times, cells were resuspended in 400–800 µL hypotonic fluorochrome solution (50 µL/mL propidium iodide and 100 µL/mL ribonuclease A (Sigma-Aldrich, Taufkirchen, Germany) in PBS (+1 mM EDTA) and incubated at 37 °C for 30 min in dark before performing FACS analysis. The propidium iodide fluorescence (FL2) of individual nuclei was measured using a FACS-Calibur cytometer (BD Biosciences, Heidelberg, Germany). Data were analyzed with the CellQuess Pro V5.2.1 software (BD Biosciences). For each condition, at least three independent experiments were performed.

### 2.8. Statistical Analyses

For COMPARE analyses, the mRNA expression values of genes of interest and log_10_IC_50_ values for acetoside were selected from the NCI database [25]. The mRNA expression has been determined by microarray analyses as reported [26,27]. COMPARE analyses were performed to produce rank-ordered lists of genes expressed in the NCI cell lines. The methodology has been described previously in detail [28]. Briefly, every gene of the NCI microarray database was ranked for similarity of its mRNA expression to the log_10_IC_50_ values for the corresponding compound. To derive COMPARE rankings, a scale index of correlations coefficients (R-values) was created. In the standard COMPARE approach, greater mRNA expression in cell lines correlate with enhanced drug resistance, whereas in reverse COMPARE analyses greater mRNA expression in cell lines indicated drug sensitivity. 

## 3. Results and Discussion

### 3.1. Cytotoxicity of Radix Scrophulariae ningpoensis

**Figure 2:**
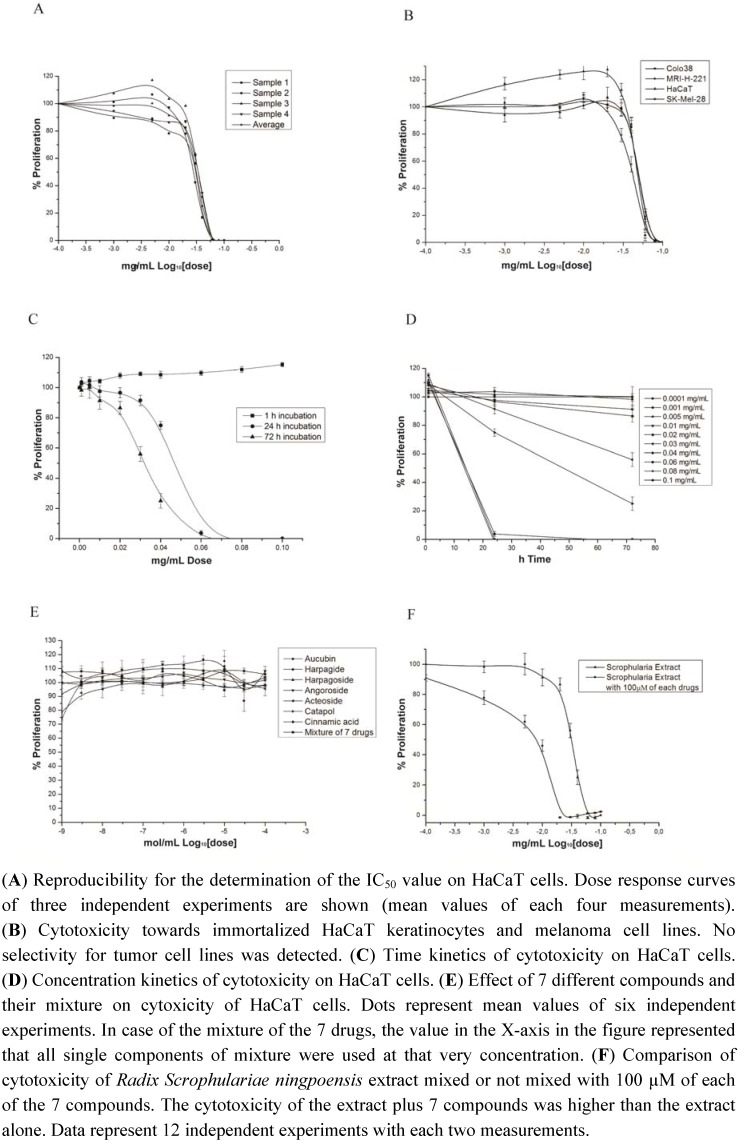
Cytotoxicity of *Radix Scrophulariae ningpoensis* as measured by XTT assay.

To test the effect of *Radix Scrophulariae ningpoensis* (RSN) extract on proliferation, we applied it to immortalized HaCaT keratinocyte cells for 72 h, and analyzed the cells by XTT test. RSN exhibited a strong growth-inhibitory effect (Figure 2A). The IC_50_ value was 0.032 mg/mL (mean value of four independent experiments). Furthermore, we investigated RSN in three different melanoma cell lines, Colo 38, SK-Mel-28 and MRI-221 (Figure 2B). RSN inhibited proliferation in a time and dose dependent manner (Figures 2C,D). However, a tumor preferred effect was not detected. Decocts containing *Scrophularia ningpoensis* have been traditionally used in China to treat dermatitis and laryngitis, but not cancer. Therefore, we decided to use HaCaT as a normal epithelial cell rather than melanoma cell lines for further experiments.

### 3.2. Enhanced Cytotoxicity of Radix Scrophularia ningpoensis

The plant’s main constituents are iridoids and phenylpropanoids [17,29]. Therefore, we included seven isolated compounds of RSN into the present study: harpagide, harpagoside, angoroside C, aucubin, catapol, acetoside, and cinnamic acid. A concentration range of 1 nM to 100 µM of each compound (with 0.1% DMSO) and a mixture of all seven drugs (1:1:1:1:1:1:1, with 0.7% DMSO) were applied to HaCaT cell for 72 h. Surprisingly, none of these phytochemicals inhibited proliferation (Figure 2E). A difference between controls (0.1% DMSO and 0.7% DMSO) was not observed.

Next, we added a mixture of 100 µM of each of these compounds to different concentrations of RSN extract and applied that mixture to HaCaT cells. This mixture did not really mimic the composition of traditionally used RSN extracts, because the proportion of each component varies considerably in the literature and a standardized mixture can hardly be defined. This is reasonable, since the plant produces various amounts of the different secondary metabolite according to differing environmental conditions during growth. As fixed proportions of the constituents can hardly be defined, we applied the highest concentrations used in prior experiments (100 µM), since no toxicity was detected at this concentration before. Interestingly, the cytotoxic activity of RSN was considerably enhanced by the compounds (Figure 2F). This result was reproducibly obtained in 12 independent experiments, indicating a synergistic effect of these compounds in RSN. However, the interaction of those components with possible other components in RSN remains unclear and needs to be further investigated.

**Figure 3:**
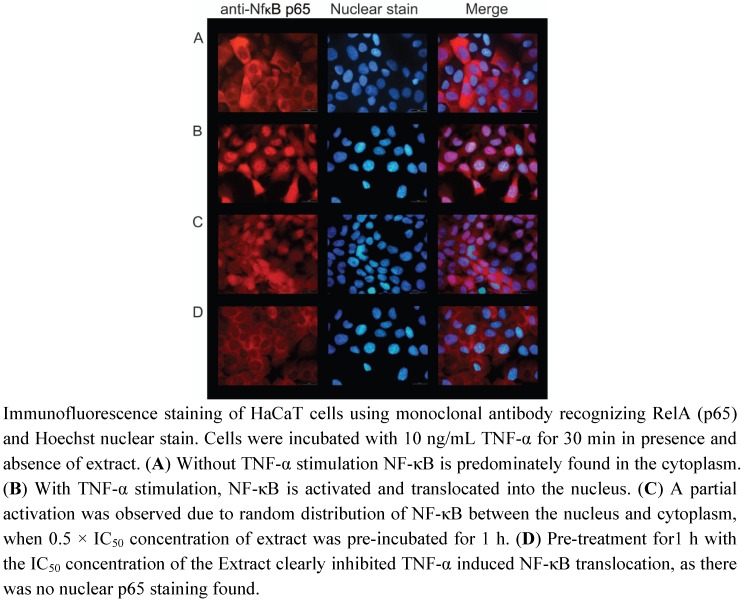
Inhibition of TNF-αinduced NF-κB activation and nuclear translocation by *Radix Scrophulariae ningpoensis* extract.

### 3.3. Inhibition of TNF-α Induced NF-κB Activation by Radix Scrophularia ningpoensis

Without TNF-α stimulation, NF-κB was predominately located in the cytoplasm of HaCaT cells, whereas NF-κB was translocated into the nucleus upon stimulation by TNF-α (Figure 3). Adding the 1 × IC_50_ concentration of RSN to cells 60 min before TNF-α stimulation resulted in a significant inhibition of NF-κB translocation into the nucleus, indicating a NF-κB activating effect of RSN (Figure 3).

**Figure 4:**
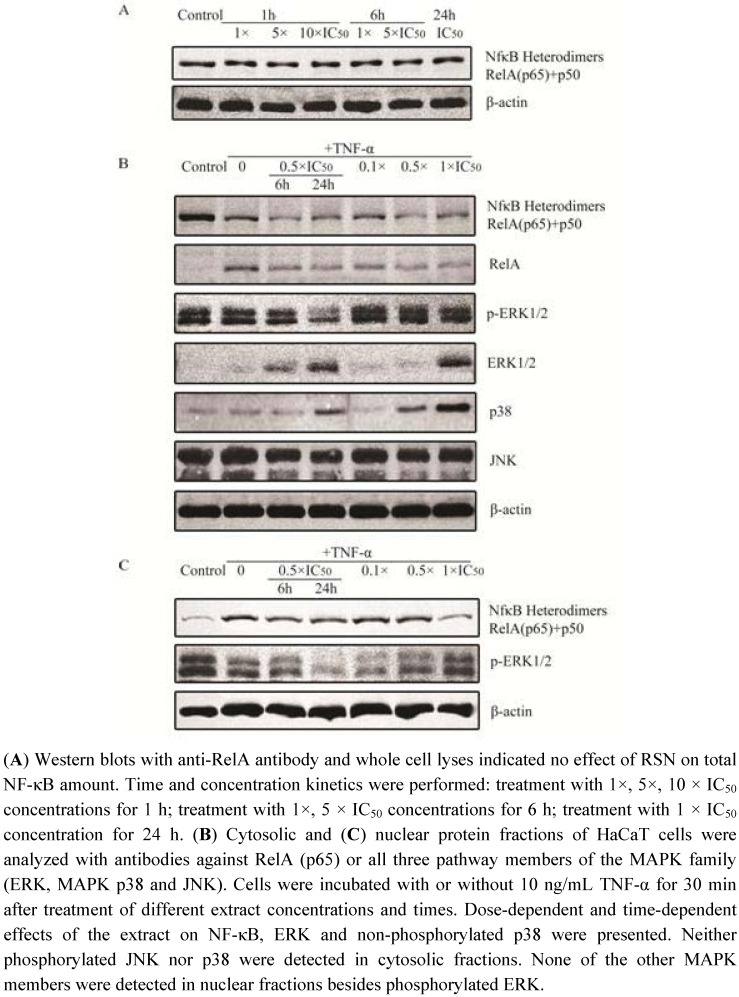
Signaling profile of the MAPK family in HaCaT cells treated with *Radix Scrophulariae ningpoensis* as measured by Western blot analysis.

To confirm these immunofluorescence findings, we investigated the subcellular localization of NF-κB by Western blot analyses. Figure 4A shows that heterodimers of p50 and p65 were found in nuclear extracts of TNF-α stimulated HaCaT cells, while RSN inhibited their translocation into the nucleus. Besides, the dimers were separated upon stimulation with TNF-α (Figure 4), as demonstrated by cytoplasma fraction samples. These data unambiguously confirmed the immunofluorescence findings (Figure 3).

Incubation with 0.1 × IC_50_ of RSN extract inferred no significant NF-κB related effect in HaCaT cells. Using 0.5 × IC_50_ of RSN resulted in a partial inhibition of NF-κB in the vast majority of cells, as demonstrated by high amounts of cells with randomly distributed NF-κB between the cell nucleus and cytoplasm (Figure 3C). This effect was enhanced upon pre-incubation for longer time intervals, e.g. 6 h or 24 h. The best inhibition of TNF-α induced NF-κB activation was achieved with 1 × IC_50_ of RSN, as seen by the highest amount of cells with loss of nuclear staining (Figure 3D).

### 3.4. Regulating effect on MAPK pathway

MAPK family members are involved in many basic biological activities, including proliferation and differentiation, by tyrosine receptors. As ERK1 and ERK2 are upstream factors for NF-κB translocation in many cell lines, we investigated the impact of RSN on all three members of the MAPK family by immunoblotting of cytoplasmic and nuclear protein fractions. As shown in Figure 4B, phosphorylated ERK was gradually decreased and non-phosphorylated ERK was gradually increased in a dose- and time-dependent manner, suggesting that ERK regulation could be one of the mechanisms of RSN to inhibit NF-κB activation.

Another interesting finding was the dose- and time-dependent increase of non-phosphorylated MAPK p38 (Figure 4B), while phosphorylated MAPK p38 was not detected (data not shown). A change in JNK expression was not detected upon RSN treatment (Figure 4B). Furthermore, we detected phosphorylated ERK1/2 in cell nuclei (Figure 4C).

### 3.5. Apoptosis and Cell Growth Inhibition Induced by Radix Scrophulariae ningpoensis

**Figure 5:**
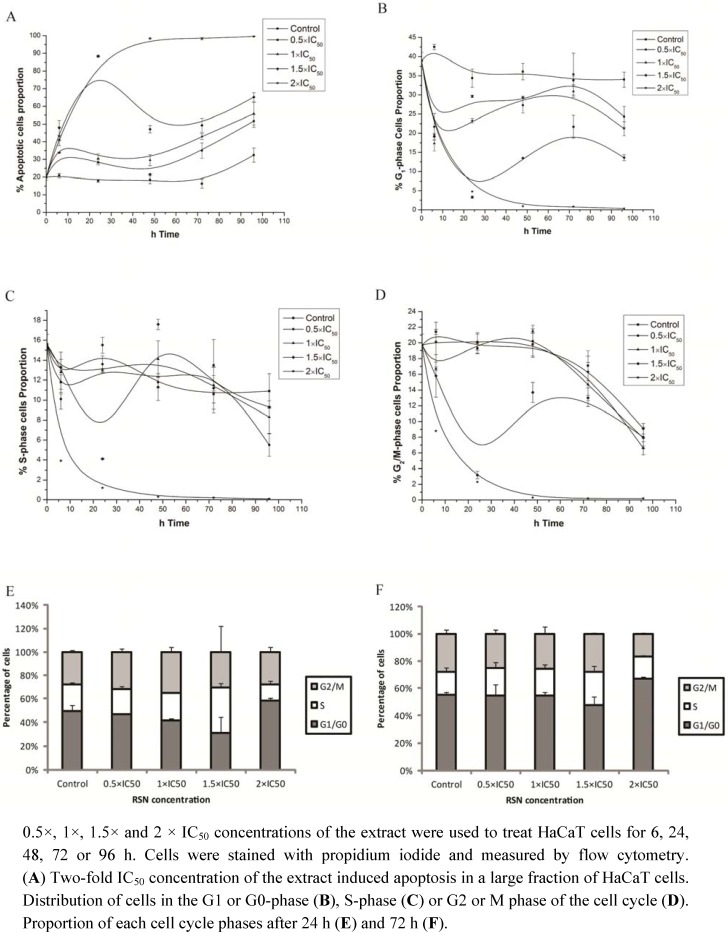
Apoptosis and cell cycle distribution of HaCaT cells treated with *Radix Scrophulariae ningpoensis* as measured by flow cytometry.

As a next step, we investigated the induction of apoptosis and cell growth arrest by RSN. Using flow cytometry, HaCaT cells were treated with 0.5-fold (0.016 mg/mL), 1-fold (0.032 mg/mL), 1.5-fold (0.048 mg/mL), and 2-fold IC_50_ concentrations (0.064 mg/mL) for 6, 24, 48, 72 and 96 h (Figure 5A). RSN (1 × IC_50_ did not induce apoptosis within 2 days. After 48 h, the apoptotic cell fraction gradually increased probably due to overpopulation and nutritional starvation. At 72 h, 1 × IC_50_ induced apoptosis in approx. 50% of cells, which proved once again the IC_50_ value and suggested that most of the cell death was due to apoptosis. At 1.5 × IC_50_, RSN significantly induced apoptosis between 6 and 24 h without further increase up to 96 h. At a concentration of 2 × IC_50_, all cells underwent apoptosis before 48 h.

Cell cycle distribution was also analyzed by flow cytometry. A cell cycle phase specifically affected by RSN was not detected (Figures 5B–F), indicating that apoptotic cells were recruited from all phases of the cell cycle.

### 3.6. Cross-Resistance of the NCI Cell Line Panel between Acetoside and Standard Drugs

To gain further insight into the activity of RSN, we searched whether the chemical constituents of *Scrophularia ningpoensis* are included in the NCI database. Acetoside was the only compound which has been tested for its cytotoxicity towards 60 tumor cell lines. Therefore, we correlated that log_10_IC_50_ values for acetoside of the 60 cell lines with the log_10_IC_50_ for more than 1,400 standard and marketed drugs in the NCI database. The log_10_IC_50_ values were selected from the NCI database As shown in Table 2, drugs with correlated with correlation coefficients of R > 0.5 are most frequently DNA-alkylating compounds, indicating firstly that the cell line panel exerts cross-resistance between acetoside and these drugs and, secondly, that acetoside might reveal a similar mode of action towards cancer cells.

**Table 2:** Correlation of log_10_IC_50_ values obtained from sulforhodamine B assays for acetoside and standard and marketed drugs as investigated by COMPARE analysis.

COMPARE Coefficient	Drug	Mode of action
0.654	cyclodisone	DNA alkylator
0.638	didemnin B	protein synthesis inhibitor
0.614	chlorozotocin	DNA alkylator
0.604	irinotecan	DNA topoisomerase I inhibitor
0.562	actinomycin D	RNA synthesis inhibitor
0.558	pipobroman	DNA alkylator
0.554	hepsulfam	DNA alkylator
0.554	melphalan	DNA alkylator
0.552	BCNU	DNA alkylator
0.552	chlorambucil	DNA alkylator
0.545	Yoshi-864	DNA alkylator
0.542	methyl-CCNU	DNA alkylator
0.532	teroxirone	DNA alkylator
0.508	aclacinomycin A	DNA intercalator

### 3.7. COMPARE and Cluster Analyses of Microarray-Based mRNA Hybridization

Next, we performed COMPARE analyses of log_10_IC_50_ values for acetoside and the transcriptomic mRNA-based expression of the 60 cell lines to produce scale indices of correlation coefficients. The mRNA expression values were selected from the NCI database. The mRNA expression was determined by microarray analyses [26,27]. We performed a standard COMPARE analysis in which cell lines most inhibited by acetoside (lowest IC_50_ values) were correlated with the lowest mRNA expression levels of genes. These genes may be considered as possible candidate genes which determine cellular resistance to acetoside. Furthermore, reverse COMPARE analyses were carried out, which correlated the most inhibited cell lines with the highest gene expression levels. Considering a COMPARE coefficient of R > 0.5 for standard COMPARE and R < −0.5 for reverse COMPARE analyses as cut-off values, the top 10 genes of both analyses whose mRNA expression correlated with acetoside are shown in Table 3. Among the genes were genes from diverse functional groups such as signal transducers (*RAC2*, *PTPRC*, *GPR68*, *SOCS1*, *RGS14*, *PTPN22*, *MAPK8*, *TSPAN6*), regulators of proliferation and cell cycle (*CDK6*, *CD53*, *MSI1*, *PTK2*), apoptosis (*PTK2*), and cell adhesion, motility, and metastasis (*KIRREL2*, *FMNL1*, *GPR68*, *PTK2*) and others (*ALDH7A1*, *DLG2*, *MPEG1*, *KIF1B*, *ATPAF1*, *ALDH1L2*).

RSN is widely used in Traditional Chinese Medicine for a broad range of diseases, including laryngitis, stress, constipation, dermatitis, neuritis, throat and respiration inflammation, as well as skin cancer [3,5]. To test its cytotoxic effects and underlying mechanisms, we performed the present study on dermatological cell lines, as little is known in this field.

Since we did not find differential sensitivities between transformed HaCaT keratinocytes and melanoma cell lines, RSN may act on cell proliferation independent of the malign state of cells. Interestingly, we found that the RSN extract but not the single isolated compounds were cytotoxic.

Neither iridoids nor phenylpropanoids (harpagide, harpagoside, aucubin, catpol, acetoside and angoroside C) inhibited cell proliferation. This result agrees with findings of other authors [5,6,7,8,9,10,11,15,17]. In contrast, these compounds are active in animal experiments [12]. Therefore, it is reasonable to assume that these phytochemicals may not affect skin cells as main target or that different mechanisms are operating *in vitro* and *in vivo*, e.g. immunomodulation by the compounds or metabolization of the substances. Obviously, the substances were indeed active, since their combination with RSN extract led to increased cytotoxicity compared to RSN extract alone. To the best of our knowledge, this is the first time that the effect of RSN was compared with single constituents. It is interesting, that the effect of RSN extract was enhanced by adding the compounds, while the same compounds themselves were not cytotoxic. The reason for this phenomenon is unknown. It may be speculated that metabolites or isomers may contribute to the synergistic effect. Further analyses are required to unravel the underlying mechanisms.

It is also the first time that *Radix Scrophulariae ningpoensis* has been investigated in human keratinocytes with respect to TNF-α induced NF-κB translocation into the nucleus. Our experiments clearly revealed dose- and time-dependent inhibition of TNF-α induced translocation of NF-κB. TNF-α stimulation induces its pro-inflammatory effects by the activation of both the AP1 signaling and the classical NF-κB pathway [30]. Interestingly, various natural compounds such as curcumin, capsaicin, resveratrol, and green tea polyphenols have previously been shown to inhibit the NF-κB pathway [31,32]. Our results are in accord with the inhibition of TNF-α induced NF-κB translocation by *Rhizoma coptidis* extract, another widely used Chinese herb [33]. It can be speculated that NF-κB activation is a common mechanism of many traditionally used herbs and their ingredients. As RSN has been applied for various inflammatory diseases in China, our finding suggested that NF-κB activation accounts for the anti-inflammatory activity of this plant. On the other hand, an inhibition of LTB4 formation was observed in animal experiments [12] and carrageenan-induced edema was reduced by harpagoside from *Scrophularia* in rats [34] pointing to other molecular modes of action.

The dose and time dependent decrease of non-phosphorylated ERK and p38 after RSN treatment suggests that RSN might affect cell proliferation and induce apoptosis. This idea was supported by propidium iodide staining and FACS analysis. However, a direct correlation of regulation of MAPK pathway and apoptosis in RSN treated cells has not been confirmed and asked for further investigation. With the present findings, we assumed that MAPK pathway regulation could be one of the mechanisms of RSN leading to cell apoptosis. In the present investigation, the induction of apoptosis by RSN has been demonstrated for the first time.

**Table 3:** Correlation of constitutive mRNA expression of genes identified by COMPARE analyses with log_10_IC_50_ values for acetoside of 60 tumor cell lines.

Symbol	COMPARE Coefficient	Pattern ID	Genbank	Name	Function
**Standard COMPARE:**					
*RAC2*	0.723	GC35722	W68830	Ras-related C3 botulinum toxin substrate 2	Signal transduction, regulates phagocytosis of apoptotic cells
				(Rho family, small GTP binding protein Rac2)	
*PTPRC*	0.706	GC30950	Y00638	Protein tyrosine phosphatase, receptor type, C	Signal transduction, T-cell activation
*CDK6*	0.697	GC168940	AW194766	Cyclin-dependent kinase 6	Cell cycle control
*GPR68*	0.695	GC74908	AI805006	G protein-coupled receptor 68	Signal transducer, metastasis suppressor in prostate cancer
*KIRREL2*	0.694	GC85039	AW025274	Kin of IRRE like 2 (Drosophila)	Cell adhesion
*FMNL1*	0.688	GC27500	AJ008112	Formin-like 1	Control of cell motility and survival of macrophages
*SOCS1*	0.682	GC53793	AB000734	Suppressor of cytokine signaling 1	Signal transduction, regulator of IL6- and LIF-signaling
*RGS14*	0.679	GC152154	AF037194	Regulator of G-protein signaling 14	Signal transduction
*PTPN22*	0.678	GC27207	AF001846	Protein tyrosine phosphatase, non-receptor	Signal transduction; negative regulator of T cell receptor signaling
				type 22 (lymphoid)	
*CD53*	0.672	GC28789	M37033	CD53 molecule	Growth regulation in hematopoietic cells
**Reverse COMPARE:**					
*MSI1*	−0.609	GC12913	H42504	Musashi homolog 1 (Drosophila) RNA	Regulates expression of target mRNAs at the translation level;
					regulates proliferation and maintenance of CNS stem cells
*MAPK8*	−0.595	GC89100	L26318	Mitogen-activated protein kinase 8	Signal transduction; activated by stress and inflammatory signals
*TSPAN6*	−0.578	GC15930	W68001	Tetraspanin 6	Signal transduction
*ALDH7A1*	−0.552	GC16889	AA024918	Aldehyde dehydrogenase 7 family, member A1	Oxidoreductase; protects cells from oxidative stress
*PTK2*	−0.551	GC18530	AA031671	PTK2 protein tyrosine kinase 2	Signal transduction: involved in cell motility, proliferation and apoptosis
*DLG2*	−0.549	GC10718	R41930	Discs, large homolog 2 (Drosophila)	Regulates surface expression of NMDA receptors
*MPEG1*	−0.534	GC18080	AA004905	Macrophage expressed 1	unknown
*KIF1B*	−0.534	GC17932	AA002163	Kinesin family member 1B	Motor for anterograde transport of mitochondria
*ATPAF1*	−0.534	GC15393	N79086	ATP synthase mitochondrial F1 complex	Role for the assembly of the mitochondrial F1-F0 complex
				assembly factor 1	
*ALDH1L2*	−0.53	GC92376	N72255	Aldehyde dehydrogenase 1 family, member L2	Oxidoreductase

Information on gene functions was taken from the OMIM database, NCI, USA [35] and from the GeneCard database of the Weizman Institute of Science, Rehovot, Israel [36].

Several animal and clinical studies have been performed with RSN. It has been described to increase the glycogen levels in mice muscles and liver [37,38]. Acetoside from *Scrophularia ningpoensis* inhibited apoptosis of hepatocytes by up-regulating the anti-apoptotic bcl-2 [18]. Other activities have been reported such as anti-oxidative effects [10], anti-inflammatory and analgesic effects [39] and platelet aggregation [12,40]. Clinical studies and case reports on *Scrophularia ningpoensis* to treat pharyngitis, dental inflammation, and inflammatory skin diseases have been published [40,41,42]. While our *in vitro* data clearly show growth inhibitory effects in immortalized keratinocytes and melanoma cells, considerable anti-cancer activity of *Scrophularia ningpoensis* decocts have clinically been found in patients with nasopharyngeal or lung cancer [44,45,46]. Further investigations should clarify whether RSN exerts specific cytotoxicity to certain cancer types, while others are not affected. Taking together, two major directions merit more detailed investigation in the future: (1) further elucidation of the signal transduction pathways involved in sensing the cytotoxic activity of RSN, especially MAP kinases and tyrosine receptors upstream of NF-κB and (2) clinical studies on the activity of RNS towards skin cancers.

The full bioactivity of RSN is determined by many phytochemicals. We have analyzed only acetoside in more detail by taking advantage of the acetoside data deposited at the NCI Drug Therapeutics Program [25], because all other compounds of RSN have not been included into this database. One has to bear in mind that the other still uninvestigated phytochemicals of RSN also exert molecular effects in tumor cells, which may contribute to synergistic interactions. A hint for the correctness of this assumption is that the addition of the major phytochemicals of RSN to the extract enhanced its cytotoxic activity. 

The correlations between acetoside and standard and marketed drugs with DNA-alkylation as major mechanism of action against cancer cells indicates firstly that the cell line panel exerts cross-resistance between acetosine and these drugs and, secondly, that acetoside might reveal a similar mode of action towards cancer cells as these drugs.

As a next step, we correlated the log_10_IC_50_ values for acetoside of 60 tumor cell lines with the microarray-based transcriptomic mRNA expression levels of this cell line panel by COMPARE analysis. This approach has been successfully used to unravel the mode of action of novel compounds [47]. Cluster and COMPARE analyses are also useful for comparing gene expression profiles with IC_50_ values for investigational drugs to identify candidate genes causing drug resistance [48] and to identify prognostic expression profiles in clinical oncology [49].

We identified genes from diverse functional groups, which were significantly associated with the response of tumor cells to acetoside, such as genes involved in signal transduction, growth regulation and cell cycle control, apoptosis, cell adhesion, motility and metastasis and others. Despite the fact that genes associated with sensitivity or resistance against acetoside were from diverse functional groups, a majority of identified genes are involved in signal transduction, regulation of proliferation, cell motility and adhesion and induction of apoptosis. This may indicate that acetoside may exert its cytotoxic activity against cancer cells by affecting signal transduction pathways. Taking the correlation of cellular response of acetoside with DNA alkylators as well as with genes involved in signal transduction into account, it can be speculated that DNA damage as primary mechanism of acetoside might subsequently lead to altered signal transduction events ultimately inhibiting proliferation and inducing apoptosis. This speculation needs to be confirmed in future studies. 

Signal transduction pathways, apoptosis, proliferation and cell cycle genes were associated with cellular response to acetoside. This nicely fits to the experimental results we obtained with RSN extract, which also exerted proliferation inhibitory, cell cycle arresting and apoptosis-inducing effects. Sohn and colleagues [50] evaluated the gene expression profile of U-87MG cells after *Scrophularia* treatment, cell cycle, proliferation, MAPK signaling pathway was affected, which is also consist with our data. In the NCI cell line panel, the cytotoxic activity of acetoside was weak (log_10_IC_50_ values in the range of −5 M to −4 M. This also fits to our data in HaCaT cells, were acetoside alone was not able to affect cellular proliferation. Nevertheless, as demonstrated by the COMPARE and hierarchical cluster analyses in our investigation, the comparison of log_10_IC_50_ values with microarray-based mRNA expression profiling provided meaningful results helpful to unravel the molecular determinants of sensitivity and resistance of tumor cells towards acetoside.

## 4. Conclusion

We investigated the bioactivity of aqueous extract of RSN and its constituents on skin derived cell lines. Cytotoxic effects of RSN extract have been detected, but failed to differentiate non-malign keratinocytes from melanoma cell lines. Seven major phytochemicals of RSN were tested in our experiments. In contrast to animal experiments, they failed to induce cytotoxicity towards HaCaT cells. However, their combination improved the effect of RSN extract, suggesting that these compounds may work synergistically with other factors. Apoptotic and anti-inflammatory effects of RNS have been detected, which is in agreement with previous clinical observations. To the best of our knowledge, we reported the influence of RSN on the MAPK and NF-κB pathways for the first time. Microarray-based Cluster and COMPARE analyses were performed with acetoside from RSN on tumor cell lines. Functional gene groups affecting cellular response towards acetoside included signal transduction, growth regulation and cell cycle control, apoptosis, cell adhesion, motility, metastasis and other pathways, which was in accord with our pathway signaling studies on HaCaT cells. Acetoside might be one active compound of RSN and DNA damage was assumed as a possible mechanism altering signal transduction events and ultimately affecting apoptosis.
